# Serum Oxidized Albumin and Cardiovascular Mortality in Normoalbuminemic Hemodialysis Patients: A Cohort Study

**DOI:** 10.1371/journal.pone.0070822

**Published:** 2013-07-29

**Authors:** Paik Seong Lim, Yachung Jeng, Ming Ying Wu, Mei-Ann Pai, Tsai-Kun Wu, Chia-San Liu, Chan Hsu Chen, Yuan-Chuan Kuo, Shiaw-Wen Chien, Hung Ping Chen

**Affiliations:** 1 Division of Renal Medicine, Tungs’ Taichung Metroharbour Hospital, Taichung, Taiwan; 2 Department of Food and Nutrition, Providence University, Taichung, Taiwan; 3 Division of Epidemiology and Biostatistics, Department of Medical Research, Tungs’ Taichung Metroharbour Hospital, Taichung, Taiwan; University of Florida, United States of America

## Abstract

**Background:**

Substantial evidence suggests that increased oxidative stress in hemodialysis (HD) patients may contribute to cardiovascular complications. Oxidative modifications of human serum albumin (HSA), the largest thiol pool in plasma, alter its biological properties and may affect its antioxidant potential in HD patients.

**Methods:**

We conducted a long-term follow-up study in a cohort of normoalbuminemic HD patients to examine the impact of redox state of serum albumin on patients’ survival by measuring the human nonmercaptoalbumin (HNA) fraction of HSA.

**Results:**

After adjusting for potential demographic, anthropometric, and clinical confounders, a positive association of HNA level with the risk of death from cardiovascular disease (CVD) and all-cause mortality was observed in normoalbuminemic HD patients. Using stratified analysis, we found a stronger association between HNA level and the risk of death from CVD and all-cause mortality in patients with pre-existing CVD.

**Conclusions:**

Serum HNA level is a positive predictor of mortality in normoalbuminemic HD patients, especially among those with pre-existing CVD. Increased oxidative stress resulting from biological changes in serum albumin levels could contribute to accelerated atherosclerosis and the development of cardiovascular disease in HD patients.

## Introduction

It has been recognized for many years that the risk of cardiovascular disease (CVD) incidence and related mortality is far greater in end-stage renal disease patients than in the general population [1], [2]. Oxidative stress has long been incriminated in the development of pathological conditions, and direct evidence for *in vivo* oxidative stress in hemodialysis (HD) patients relied almost entirely on the measurement of oxidative by-products of circulatory biomolecules. Proteins are extremely susceptible to oxidative stress, but studies on the detection of oxidative modified proteins have not been extensive. Recently, the measurement of markers for protein oxidation, such as advanced protein oxidation products (AOPP) or carbonyl contents, has been applied to assess the levels of oxidative stress in dialysis patients [3], [4].

Human serum albumin (HSA) is the most abundant soluble protein in the body and is generally considered to act as a multifunctional transport protein. Apart from contributing to plasma oncotic pressure, albumin plays an important role as a versatile transporter for numerous molecules, including hormones, several important classes of drugs, and endogenous toxins. Recently, accumulating evidence suggests that albumin also has significant antioxidant activity [5]–[9]. These studies have suggested that albumin, which is the largest thiol pool in plasma, contributes to the protective mechanism for maintaining cellular and regulatory long-lived proteins. The human isoform of serum albumin is a 67-kD protein containing 585 amino acids, including 35 cysteine (Cys) residues. Among the 35 cysteine residues, 34 are involved in the formation of 17 stabilizing disulfide bonds [6]. Several studies of the antioxidant activity of HSA in different clinical situations have focused on the importance of the single, free sulfhydryl group at amino acid position 34 (Cys-34, the position is measured from the N-terminus) [10], [11]. Chemically, human serum albumin (HSA) can be further divided into at least three forms according to the redox state of Cys-34: unoxidized [human mercaptoalbumin (HMA)], reversibly oxidized [human nonmercaptoalbumin-1 (HNA-1)], and strongly oxidized [human nonmercaptoalbumin-2 (HNA-2)]. The one free sulfhydryl group at Cys-34 in HMA forms the largest fraction of free sulfhydryl in the serum. In healthy adults, about 70%–80% of Cys-34 in albumin belongs to this form. However, HNA consists of at least three types of compound forms, and the major HNA compounds are complexes of disulfide and cysteine [HNA (Cys)] or glutathione [HNA (Glut)]. The other is an oxidation product with an oxidation number higher than that of mixed disulfides [HNA (Oxi)], which is a minor species in extracellular fluids [12]. As the major plasma protein, albumin is predicted to be a target of modification during oxidative stress, and oxidative damage of albumin may impair its function. This is particularly relevant in disease states such as uremia, in which tissues are known to be exposed to continuous oxidative stress [13]. The fraction of HNA is negatively correlated with creatinine clearance in patients with renal dysfunction [14]. A direct protective effect of albumin has been indicated from many epidemiological studies [15]–[17]. The results of *in vitro* experiments also lend support to this hypothesis because they show that albumin protects human low-density lipoproteins against copper-mediated oxidation and blood against hemodialysis by free radicals [7], [9], [18].

Serum albumin level may reflect the states of nutrition and inflammation in HD patients [16], [19]. Many previous studies have shown that hypoalbuminemia is one of the strongest predictors of death in maintenance HD patients [16], [20], [21]. However, in daily practice, we have observed a much higher mortality rate than what could be explained by hypoalbuminemia alone. We have also observed that a considerable number of normoalbuminemic HD patients die from CVD. This motivated us to revisit the risk factors for mortality in this patient group. Recent mounting evidence has suggested that qualitative changes in serum albumin are found in these patients [9], [22]–[24]. Early studies reported that the protective activity of HSA against erythrocyte membrane lipid peroxidation is decreased in patients with renal failure [7], [9] and that this noxious state can be ameliorated by administration of HSA from healthy subjects [9]. Therefore, it is plausible to assume that there is a potential association between the scavenging capabilities of HSA and the long-term complications in HD patients with particular cardiovascular complications. This study aimed to investigate the association between the redox state of serum albumin and mortality in HD patients with normal albumin levels.

## Materials and Methods

### Study Design and Participant Enrollment

We conducted a prospective observational study of 249 prevalent hemodialysis patients in June 2006. The cohort was derived from a regional hospital in the coastal area of central western Taiwan. All participants were followed from enrollment to death or to the end of the study (June 30, 2009), whichever came first. Among the 249 patients recruited, one withdrew and was excluded from the analysis.

Patients who received HD for at least 3 months and had normal serum albumin levels (≥3.5 g/dL) for the previous 6 months were included. Patients with an overt active inflammatory state, infection, autoimmune disease, liver cirrhosis, severe heart failure, malignancy, and those receiving immunosuppressive therapy were excluded.

The primary outcome measures were CVD mortality and overall mortality at 6.5 years of follow-up. CVD mortality was defined as death from coronary heart disease, congestive heart failure, sudden death, or stroke. The survival status and cause of death were ascertained from chart review or confirmed by case managers. The study was conducted in accordance with the Declaration of Helsinki and its amendments. The protocol was approved by the Institutional Review Board of Tungs’ Taichung MetroHarbor Hospital, and written informed consents were obtained from all patients before enrollment.

### Baseline Measurements

Baseline data, such as demographic factors, dialysis vintage, previous cardiovascular events (including a pre-existing coronary event, stroke, peripheral vascular disease, or positive coronary angiographic studies), the albumin redox state, and significant clinical indices of diabetes mellitus (DM) and CVD, were collected at the start of the study (Table 1). All patients were dialyzed thrice weekly with a polysulfone hollow-fiber dialyzer (Fresenius Polysulfone®, Fresenius Medical Care; Bad Homburg, Germany) with 1.8 m^2^ and 2.0 m^2^ surface areas, and the bicarbonate-based dialysate was delivered at a bicarbonate level of 34 mEq/L.

**Table 1:** The characteristics of study samples according to HNA level and the baseline cardiovascular disease history.

	With CVD history		Without CVD history			
Variables	HNA≤51.16%	HNA>51.16%	HNA≤51.16%	HNA>51.16%	*P*-value	
	*Frequency (percentage)*					
Sex					0.1428	
Male	37(45%)	19(51%)	59(57%)	9(36%)		
Female	45(55%)	18(49%)	45(43%)	16(64%)		
Diabetes mellitus					0.4517	
Yes	54(66%)	27(73%)	38(37%)	8(32%)		
No	28(34%)	10(27%)	66(63%)	17(68%)		
Hypertension^a^					0.1135	
Yes	69(85%)	35(95%)	66(63%)	17(68%)		
No	12(15%)	2(5%)	38(37%)	8(32%)		
	*Mean±standard deviation*					
HNA fraction (10%)	4.0±0.7	6.0±0.7	3.8±0.6	5.9±0.7	<.0001*	
HMA fraction (10%)	6.0±0.7	4.0±0.8	6.2±0.6	4.1±0.7	<.0001*	
HNA-1 fraction (10%)	3.7±0.7	5.7±0.7	3.6±0.6	5.6±0.7	<.0001*	
HNA-2 fraction (%)	2.2±0.3	2.8±0.3	2.2±0.3	2.8±0.4	<.0001*	
Dialysis vintage (yr)	3.9±4.3^(1)^	3.1±2.4^(1)^	3.2±4.1	4.0±4.9^(1)^	0.6408	
Age at enrollment (yr)	67.1±9.9	68.7±11.7	62.7±13.1	60.0±15.9	0.0551	
Body height (cm)	158.8±7.7	159.2±8.6	160.4±8.1	156.2±9.5	0.4128	
Body weight (kg)	58.3±10.9	59.8±11.4	60.6±11.7	54.5±10.1	0.6171	
BMI (kg/m^2^)	23.1±3.7	23.6±3.9	23.5±3.6	22.4±4.1	0.7877	
DBP (mmHg)	76.1±13.1	80.4±11.3^(1)^	77.1±11.5	81.4±12.7	0.1452	
SBP (mmHg)	139.3±25.3	143.3±20.8^(1)^	136.5±22.8	136.8±19.0	0.8440	
hsCRP (mg/L)	4.7±4.2	5.0±2.8	3.3±2.7	3.0±2.4	0.0259*	
Hemoglobin (g/dL)	10.7±1.2	10.4±1.0	10.8±1.2	11.1±1.4	0.0297*	
Hematocrit (%)	32.9±3.4	32.2±3.1	33.0±3.4	33.7±4.2	0.0494*	
Ferritin (ng/mL)	611.9±323.5	644.2±380.8	581.0±294.8	584.4±243.1	0.2068	
WBC (/µL)	7201±1876	7270±1819	6536±1768	6512±1557	0.4329	
FBS (g/dL)	120.7±43.5	134.8±50.1	106.2±38.2	98.4±21.5	0.3617	
Triglyceride (mg/dL)	147.0±93.7	172.3±110.5	137.5±82.7	126.9±64.2	0.1359	
Total cholesterol (mg/dL)	179.4±35.2	166.7±34.3	174.1±39.8	182.2±39.3	0.6471	
HDL-C (mg/dL)	45.8±15.1^(1)^	38.8±13.3	47.1±14.6^(3)^	56.6±20.9^(1)^	0.1282	
LDL-C (mg/dL)	104.4±31.8^(1)^	93.5±29.5	99.7±34.1^(3)^	101.7±31.2^(1)^	0.3764	
HDL-C/Chol	0.3±0.1^(1)^	0.2±0.1	0.3±0.1^(3)^	0.3±0.1^(1)^	0.2439	
LDL-C/Chol	0.6±0.1^(1)^	0.6±0.1	0.6±0.1^(3)^	0.6±0.1^(1)^	0.2983	

Note: The superscript numbers in parentheses indicate the number of missing values.

* A significant association indicated by Spearman’s correlation test.

Abbreviations: CVD, cardiovascular disease; HNA, human non-mercaptoalbumin; HMA, human mercaptoalbumin; HNA1, human non-mercaptoalbumin-1; HNA2, human non-mercaptoalbumin-2; BMI, body mass index; DBP, diastolic blood pressure; SBP, systolic blood pressure; hsCRP, high-sensitive C-reactive protein; WBC, white blood cells; ferritin, serum ferritin; FBS, fasting blood sugar; HDL-C, high density lipoprotein cholesterol; LDL-C, low density lipoprotein cholesterol; HDL-C/Chol, the HDL-C to total cholesterol ratio; LDL-C/Chol, the LDL-C to total cholesterol ratio.

a One patient with preexisting CVD and DM has unknown HTN status.

### Biochemical Determinations

Blood samples for laboratory testing were drawn from the venous end of a vascular access at the beginning of the hemodialysis session and then stored at −80°C until time of analysis. The high sensitive C-reactive protein (hsCRP) levels were determined by a commercial immunoturbidimetric assay using a Hitachi autoanalyzer (model 7170). The detection limit and interval for CRP was 0.1 mg/L and 0.1–500.0 mg/L, respectively. The baseline serum albumin was measured by the bromocresol green method on a Hitachi autoanalyzer (model 7170). Serum levels of cholesterol, triglyceride, and low- and high-density lipoprotein cholesterol were determined by standard laboratory methods.

### Measurement of the Albumin Redox State

Measurement of the albumin redox state was performed using the high-performance liquid chromatography (HPLC) method reported previously with some modifications [11], [12], [25], [26]. The HPLC-fluorescence detection (HPLC-FD) system consisted of an AS-8010 autosampler (injection volume, 2 mL per specimen; Tosoh, Tokyo, Japan) and a Model FS-8000 fluorescence detector (excitation wavelength, 280 nm; emission wavelength, 340 nm) with a CCPM double-plunger pump (Tosoh) in conjunction with a SC-8020 system controller (Tosoh). A Shodex-Asahipak ES-502N 7C column [Showa Denko, Tokyo, Japan; 10×0.76 cm (inner diameter), dimethylaminoethyl-form for ion-exchange HPLC, column temperature, 35°C±0.5°C] or in some instances two Asahipak GS-520H columns [Asahi Chemical Industry; Kawasaki, Japan; 25×0.75 cm (inner diameter) maintained at 32°C] were used. Linear gradient elution was carried out with an ethanol level increasing from 0% to 5% in 0.05 M sodium acetate and 0.40 M sodium sulfate buffer (pH 4.85; acetate–sulfate buffer) at a flow rate of 1.0 mL/min. Deaeration of the buffer solution was performed by helium bubbling. Based on the HPLC profiles of HSA obtained from these procedures, the values for each fraction were subjected to numerical curve fitting, and the fractions of HMA, HNA-1, and HNA-2 to total HSA were calculated.

### Statistical Analysis

The descriptive statistics were expressed as mean ± standard deviation (SD) for continuous variables and frequency (percentage) for categorical variables. The redox state of serum albumin was quantified by the fraction of HNA in total HSA. To better illustrate the results, HNA level was redefined as a dichotomous variable with a value of 0 if the fraction was higher than the third quartile and 1 if otherwise. Descriptive statistics were stratified according to the dichotomous HNA level. Spearman’s test and the log-rank test were applied as appropriate to assess bivariate associations among the HNA fraction, survival outcomes, and potential confounders. The associations between the HNA fraction and overall mortality as well as CVD mortality were assessed by Cox proportional hazards models, which were also applied to assess the effects of other variables on both mortalities. To analyze the risk factor effect on CVD death, both non-CVD deaths and the end of study were treated as noninformative censoring events. The final models were constructed by stepwise selection, with criteria for entry and stay in model set to levels of <0.2 and <0.1, respectively. The differences in Akaike’s Information Criterion were further used to evaluate whether significant increases in goodness-of-fit could be gained from reserving covariates of *p*-values between 0.05 and 0.1. The hazard ratio (HR), 95% confidence interval (95% CI), and *p*-value from the Cox regression model analysis were reported. Throughout this paper, a statistically significant association was considered when the *p*-value is <0.05. Stratified analysis was performed according to baseline CVD history status (with vs. without) to investigate the clinical significance of redox state measurements for HD patients. All statistical analyses were performed using the SAS statistical software package (SAS Institute Inc.; Cary, NC, USA).

## Results

### Descriptive Analyses of the Study Cohort

The 248 HD patients in this study had a mean (SD) HNA level of 43.9% (11.2%). The mean follow-up time was 4.9 (2.2) years with a range of 3 months to 6.5 years. The mean age was 65 (13) years, the mean body mass index (BMI) was 23.3 (3.7) kg/m^2^, and 50% of the patients were men. The mean serum albumin level was 4.1 (0.3) g/dL. The dialysis vintage ranged from 3 months to 21.7 years, with a mean of 3.5 years and median of 2.1 years. The three quartiles of HNA measurement for patients with a CVD history at baseline were 36.96, 44.86, and 53.67, with range 23.99–84.27; these values for patients without a CVD history at baseline were 34.76, 41.08, and 48.98, with a range of 22.88–79.25.

Spearman’s correlation tests showed that the participants with higher HNA measurements were more likely to have pre-existing CVD, high hsCRP levels, and low hemoglobin and serum albumin levels (the respective *p*-values were 0.0077, 0.0259, 0.0297, and 0.0494). Patients with high HNA levels also exhibited higher fractions of HNA-1 and HNA-2. Detailed clinical characteristics of the participants are summarized in Table 1.

### Survival Analysis for the Entire Study Cohort

**Figure 1:**
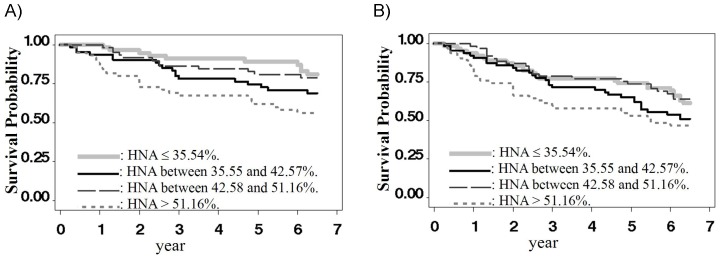
The Kaplan-Meier estimates for survival outcomes. The survival functions of (A) the time to death from cardiovascular disease, and (B) the time to death from any causes according to HNA levels. The corresponding log-rank test p-values are 0.0038 and 0.0747.

One hundred ten patients died during the 6.5-year follow-up period. Among these patients, 65 (41%) died as a result of causes other than CVD, such as sepsis or malignancy. The detailed distribution of survival outcomes by HNA quartiles is displayed in Table 2. The crude survival probabilities were 90.32%, 80.65%, 71.77%, 70.97%, 66.53%, and 59.27% by the end of the first, second, third, fourth, fifth, and sixth years, respectively. The estimated survival functions of the time to death from CVD and all causes were stratified by HNA quartiles and illustrated in Figure 1. Patients with a serum HNA fraction above the upper quartile (those with HNA>51.16%) had a significantly higher CVD mortality than other patients after the first year, but the difference in overall mortality was not statistically significant (*p*-value = 0.0747).

**Table 2:** Summary of 6.5 year follow-up according to HNA level and the baseline cardiovascular disease history.

		HNA Quartiles				
Variables		Q1		Q2	Q3	Q4
		(≤35.54%)		(35.55–42.57%)	(42.58–51.16%)	(>51.16%)
*Patients with known CVD history at baseline* (*n* = 119)						
*Survival status*	*frequency (percentage)*					
Die of CVD	7(28%)		12(52%)		12(35%)	22(59%)
Die of other causes	8(32%)		3(13%)		5(15%)	7(19%)
Survive	10(40%)		8(35%)		17(50%)	8(22%)
*Survival time (yr)* †	*mean*±*SD*					
Die of CVD	3.9±2.4		2.8±1.9		2.8±1.7	2.2±1.8
Die of other causes	2.9±2.3		4.3±2.1		4.7±2.1	2.1±1.8
*Patients without known CVD history at baseline* (*n* = 129)						
*Survival status*	*frequency (percentage)*					
Die of CVD	3(8%)		6(15%)		0(0%)	3(12%)
Die of other causes	6(16%)		10(25%)		5(19%)	1(4%)
Survive	28(76%)		24(60%)		22(81%)	21(84%)
*Survival time (yr)* †	*mean*±*SD*					
Die of CVD	3.7±2.1		2.7±1.9		–±–	3.4±1.8
Die of other causes	3.0±2.3		3.6±2.0		2.9±1.9	1.0±–

Abbreviations: CVD, cardiovascular disease; HNA, human non-mercaptoalbumin.

† All the survivors were followed for 6.5 years.

The crude HRs of death from CVD and all causes for all variables are listed in Table 3, along with the 95% CIs. Since the fractions of HNA and HMA in total HSA sum up to 100% [13], the HR of mortalities associated with HMA is the reciprocal of that associated with HNA, but the statistical significance remains the same. A high level of HNA or HNA-1 or a history of CVD, DM, or hypertension was positively correlated with both mortalities. There was a statistically significant survival benefit in patients who were younger, who had higher body weight, who had higher BMI, high-density lipoprotein cholesterol (HDL-C) or HDL-C to total cholesterol ratio (HDL-C/Chol), or who had lower levels of hsCRP or FBS. The results of multiple Cox regression analysis are displayed in Table 4.

**Table 3:** The unadjusted hazard ratios for survival outcomes.

Cause of death	CVD			All causes		
Variable	HR	95% CI	P-value	HR	95% CI	P-value
HNA fraction (10%)	1.44	(1.178, 1.762)	0.0004*	1.20	(1.015, 1.409)	0.0328*
HNA>51.16% *vs*. ≤51.15%	2.27	(1.379, 3.748)	0.0013*	1.56	(1.039, 2.35)	0.032*
HMA fraction (10%)	0.69	(0.568, 0.849)	0.0004*	0.84	(0.709, 0.985)	0.0324*
HNA-1 fraction (10%)	1.46	(1.187, 1.796)	0.0003*	1.20	(1.017, 1.426)	0.031*
HNA-2 fraction (%)	1.89	(1.074, 3.308)	0.0273*	1.29	(0.816, 2.032)	0.2777
pre-existing CVD (y *vs.* n)	6.42	(3.426,12.037)	<.0001*	3.29	(2.195, 4.944)	<.0001*
Sex (male *vs.* female)	0.95	(0.582, 1.54 )	0.8268	1.10	(0.754, 1.594)	0.6313
Diabetes mellitus (y *vs.* n)	2.84	(1.647, 4.886)	0.0002*	1.85	(1.253, 2.716)	0.0019*
Hypertension (y *vs.* n)	2.97	(1.355, 6.503)	0.0066*	1.84	(1.112, 3.055)	0.0177*
Dialysis vintage (yr)	0.95	(0.885, 1.016)	0.1286	0.95	(0.897, 0.997)	0.0393*
Age at enrollment (yr)	1.03	(1.008, 1.051)	0.0078*	1.05	(1.03, 1.065)	<.0001*
Body height (cm)	0.99	(0.962, 1.02 )	0.5468	1.00	(0.975, 1.02)	0.7975
Body weight (kg)	0.98	(0.955, 1)	0.0455*	0.98	(0.965, 0.999)	0.0392*
BMI (kg/m2)	0.93	(0.871, 1.002)	0.0562	0.94	(0.892, 0.993)	0.0259*
DBP (mmHg)	1.00	(0.981, 1.021)	0.9508	0.99	(0.975, 1.005)	0.1767
SBP (mmHg)	1.01	(0.999, 1.022)	0.07	1.00	(0.995, 1.012)	0.384
hsCRP (mg/L)	1.14	(1.076, 1.202)	<.0001*	1.16	(1.117, 1.208)	<.0001*
Hemoglobin (g/dL)	0.94	(0.771, 1.154)	0.572	0.94	(0.802, 1.093)	0.4062
Hematocrit (%)	1	(0.935, 1.072)	0.9663	0.99	(0.937, 1.042)	0.6551
Ferritin (g/dL)	1	(0.999, 1.001)	0.7888	1	(1, 1.001)	0.4952
WBC (/µL)	1	(1, 1)	0.1478	1	(1, 1)	0.0524
FBS (mg/dL)	1.01	(1.002, 1.012)	0.0053*	1.01	(1.003, 1.011)	0.0006*
Triglyceride (mg/dL)	1.00	(0.999, 1.004)	0.132	1.00	(0.999, 1.003)	0.3034
Total cholesterol (mg/dL)	1.00	(0.993, 1.006)	0.8642	1.00	(0.993, 1.003)	0.3879
HDL-C (mg/dL)	0.98	(0.957, 0.993)	0.007*	0.97	(0.958, 0.986)	0.0001*
LDL-C (mg/dL)	1.00	(0.993, 1.009)	0.7994	1.00	(0.995, 1.007)	0.8041
HDL-C/Chol	0.01	(0.001, 0.26)	0.0048*	0.02	(0.002, 0.17)	0.0006*
LDL-C/Chol	3.12	(0.191,50.937)	0.4242	6.75	(0.76,59.847)	0.0865

Note: See the footnotes of Table 1 for the abbreviations. The asterisks “*” indicate variables with significant effect on death hazard rate at a 0.05 level.

**Table 4:** The adjusted hazard ratios for survival outcomes.

Cause of death	Cardiovascular disease			All causes		
Variable	HR	95% CI	P-value	HR	95% CI	P-value
*All patients* (*n* = 248)						
Pre-existing CVD	4.61	(2.380, 8.945)	<.0001*	2.34	(1.511, 3.624)	0.0001*
HNA>51.16% vs. ow.	2.22	(1.342, 3.685)	0.0019*	1.57	(1.037, 2.382)	0.0330*
hsCRP (mg/L)	1.10	(1.033, 1.168)	0.0026*	1.11	(1.058, 1.164)	<.0001*
Age at enrollment (yr)	–	–	–	1.03	(1.01, 1.047)	0.0022*
DM (y vs. n)	2.04	(1.155, 3.600)	0.0140*	–	–	–
BMI (kg/m^2^)	0.91	(0.843, 0.975)	0.0078*	0.91	(0.857, 0.958)	0.0005*
Vintage (yr)	–	–	–	0.93	(0.873, 0.979)	0.0076*
HDL-C/Chol	–	–	–	0.09	(0.007, 1.071)	0.0565
*Patients with known CVD history at baseline* (*n* = 119)						
HNA>51.16% vs. ow.	2.55	(1.456, 4.467)	0.0011*	1.78	(1.115, 2.846)	0.0156*
hsCRP (mg/L)	1.10	(1.032, 1.175)	0.0037*	1.14	(1.083, 1.196)	<.0001*
DM (y vs. n)	1.99	(1.042, 3.813)	0.0370*	–	–	–
BMI (kg/m2)	0.90	(0.831, 0.978)	0.0124*	–	–	–
Hypertension (y vs. n)	–	–	–	3.93	(1.402,11.042)	0.0093*
*Patients without known CVD history at baseline* (*n* = 129)						
hsCRP (mg/L)	–	–	–	1.21	(1.1, 1.34)	0.0001*
Ferritin (ng/mL)	0.997	(0.994, 1)	0.0386*	–	–	–
Age at enrollment (yr)	–	–	–	1.08	(1.05, 1.119)	<.0001*
FBS (g/dL)	–	–	–	1.01	(1.004, 1.023)	0.0039*

Note: See the footnotes of Table 1 and Table 2 for the abbreviations.

The association of CVD death risk with HNA, hsCRP, baseline DM status, and BMI remained significant after adjusting for history of previous CVD. The CVD death risk of patients with a high HNA level (>51.16%) was estimated to be 2.22 times higher than that of patients with a low HNA level. The HR of all-cause death for patients with a high HNA level versus those with a low HNA level was 1.57, after adjusting for the effect of baseline CVD history status, hsCRP level, age at enrollment, BMI, dialysis vintage, and HDL-C/Chol. The estimated HRs, 95% CIs, and *p*-values of these confounders are shown in Table 4.

### Stratified Survival Analysis According to the Baseline CVD Status

The results of stratified analysis are shown in Table 4. A high HNA level was associated with high CVD death risk for patients with a known CVD history at baseline. The estimated HRs was 2.55 and 2.16 (95% CI, 1.47–4.47 and 1.31–3.56) for the risk of death from CVD and all causes, respectively. Such an association was not found in patients without a known CVD history at baseline. In this subset of patients, a high ferritin level was associated with a low risk of death from CVD, whereas low hsCRP level, young age, and low fasting blood sugar level were associated with decreased risk of death from all causes.

## Discussion

This prospective study investigated the association between the redox state of human serum albumin and mortality in HD patients. The redox state of HSA was quantified by measuring the fraction of HNA to total serum HSA using HPLC [13]. Two main findings were observed. First, the low serum HNA fraction was associated with a survival benefit in normoalbuminemic HD patients. Second, stratified analysis revealed a strong association between HNA and the risk of death from CVD and all causes in patients with pre-existing CVD. Conventional independent predictors of the mortality of HD patients, such as high hsCRP level, old age, the presence of DM or hypertension, low BMI, low HDL-C/Chol, and poor glycemic control, were also observed in our study.

In our HD patients, substantial oxidative modification of serum albumin leads to increases in the HNA-1 and HNA-2 fractions. This finding is *comparable* with *results obtained in previous* studies [13], [14]. These earlier studies have shown the alteration of redox state of serum albumin in CKD patients, but its association with clinical outcomes has rarely been reported. Terawaki *et al.* [27] reported the association of serum albumin redox state and incidence of serious CVD. Nevertheless, our data extend previously published studies in terms of cohort size as well as follow-up time and, for the first time, reveal that an increase in the serum HNA fraction is strongly associated with high CVD mortality in normoalbuminemic HD patients, especially in those having a pre-existing CVD history. In this study, we demonstrated that the redox state of HSA is a positive predictor of CVD mortality in normoalbuminemic HD patients, especially in patients with a known CVD history. Using serial HNA surveillance measurements, rather than a single baseline observation, to predict the risk of death would be worthy of future study and this would be helpful to better assess the qualitative changes in serum albumin in CKD patients.

Previous studies showed that adequate hemodialysis significantly rescue serum albumin reduction and considerably decreased the HNA fraction after a single HD session [13], [28]. Other studies observed that the post-dialytic HMA fraction showed a stronger association with serious CVD incidence and mortality than the pre-dialytic HMA fraction in HD patients without a CVD history at baseline [27]. Because the post-HD HSA-redox state was not evaluated in this study, its association with CVD mortality and overall mortality cannot be assessed. Nonetheless, this limitation does not impact the clinical value of this study. With reference to previous observations by Terawaki *et al.* [27] and Soejima *et al.* [13], the pre-HD and post-HD HSA-redox states exhibit a coherent pattern of association analysis and do not fluctuate over 10% in most patients. In our view, pre-HD HSA levels more closely reflect the redox state of patients because this measurement revealed the burden of the pro-oxidant during the interdialytic period. Post-HD measurements merely reflect how a dialysis session could partially restore the redox state of albumin. More importantly, the mechanisms underlying intradialytic mercapt–nonmercapt conversions remain unclear.

For many years, we have been aware that hypoalbuminemia is a predictor of cardiovascular morbidity [29] and mortality [30] in HD patients. It is likely that the cause of hypoalbuminemia, rather than albumin levels *per se*, drives increased morbidity in uremic patients. In some previous studies, an association was found among hypoalbuminemia, oxidative stress, and inflammation in HD patients [19], [31]. One recent study demonstrated a direct molecular mechanism linking oxidative stress and hypoalbuminemia [24]. Hence, oxidative stress, as reflected by hypoalbuminemia, may be casually related to clinical outcomes in HD patients.

Albumin, being the most abundant plasma protein, forms the largest fraction of reactive sulfhydryl (free thiol groups) and acts as an effective antioxidant system in plasma [32]. HSA contains one reduced cysteine residue, Cys-34. It has been reported that HSA Cys-34 is highly accessible to reactive oxygen species such as hydrogen peroxide (H_2_O_2_) [33], [34] and carbon-centered free radicals [35], as well as other oxidizing agents such as nitric oxide (NO) [19], [24], [31], [35] and peroxynitrite (ONOO^−^) [19], [36]. Undoubtedly, the mercapt–nonmercapt conversion (intermolecular sulfhydryl–disulfide exchange reaction) of serum albumin is an effective antioxidant system in extracellular fluids [37]. However, oxidation of Cys-34 in HSA leads to the formation of sulfenic acid (RSOH), which is further oxidized to sulfinic (RSO_2_H) or the sulfonic acid form (RSO_3_H) [29], [31], [34], [36]. This oxidative process leads to conformational alterations of HSA, resulting in impaired or decreased antioxidant activity [9], [38], [39]. Hence, an increase in the oxidized form of HSA (HNA fraction) may aggravate the existing oxidative status of HD patients. In the absence of the protective role of biologically and chemically active albumin, HD patients are exposed to oxidative damage by reactive free radicals in extracellular fluids, which may contribute to the development and aggravation of cardiovascular and atherosclerotic complications [40], [41].

Finally, the patients included in this study had normal plasma albumin levels and showed no clinical evidence of malnutrition. Therefore, an impact of the oxidized fraction of albumin was observed even in “well-nourished” patients with normoalbuminemia. In addition, our findings are of interest because they provide an explanation for the link between serum albumin levels and CVD mortality. This view is supported by several previous studies that demonstrated that the burden of oxidative modified molecules in dialysis patients is associated with CVD and overall mortality [42]–[44].

In summary, the results of this prospective study suggest that the serum HNA level is a positive predictor of mortality in normoalbuminemic HD patients, especially among those with pre-existing CVD. Alterations of the redox state of serum albumin with impaired scavenging activity could contribute to accelerated atherosclerosis and the development of cardiovascular disease in HD patients.

## References

[1] Foley RN, Parfrey PS, Sarnak MJ (1998) Clinical epidemiology of cardiovascular disease in chronic renal disease. Am J Kidney Dis (suppl 3): S112–S119. 10.1053/ajkd.1998.v32.pm9820470 9820470

[2] Levin A, Singer J, Thompson CR, Ross H, Lewis M (1996) Prevalent LVH in the predialysis population: Identifying opportunities for intervention. Am J Kidney Dis 27: 347–354. 8604703 10.1016/s0272-6386(96)90357-1

[3] Descamps-Latscha B, Witko-Sarsat V (2001) Importance of oxidatively modified proteins in chronic renal failure. Kidney Int (Suppl 78): S108–S113. 10.1046/j.1523-1755.2001.59780108.x 11168994

[4] Daschner M, Lenhartz H, Bötticher D, Schaefer F, Wollschläger M, el al (1996) Influence of dialysis on plasma lipid peroxidation products and antioxidant levels. Kidney Int 50: 1268–1272. 8887287 10.1038/ki.1996.437

[5] Halliwell B (1988) Albumin - an important extracellular antioxidant? Biochem Pharmacol 37: 569–571. 3277637 10.1016/0006-2952(88)90126-8

[6] Peters T Jr (1996) All about Albumin: Biochemistry, Genetics, and Medical Applications. San Diego, CA: Academic Press. 51–54.

[7] Bourdon E, Loreau N, Blache D (1999) Glucose and free radicals impair the antioxidant properties of serum albumin. FASEB J 13: 233–244. 9973311 10.1096/fasebj.13.2.233

[8] Dean RT, Hunt JV, Grant AJ, Yamamoto Y, Niki E (1991) Free radical damage to proteins: The influence of the relative localization of radical generation, antioxidants, and target proteins. Free Radic Biol Med 11: 161–168. 1937134 10.1016/0891-5849(91)90167-2

[9] Lim PS, Cheng YM, Yang SM (2007) Impairments of the biological properties of serum albumin in patients on haemodialysis. Nephrology 12: 18–24. 17295656 10.1111/j.1440-1797.2006.00745.x

[10] Cha MK, Kim IH (1996) Glutathione-linked thiol peroxidase activity of human serum albumin. Biochem Biophys Res Commun 222: 619–625. 8670254 10.1006/bbrc.1996.0793

[11] Era S, Kuwata K, Imai H, Nakamura K, Hayashi T, et al (1995) Age-related change in redox state of human serum albumin. Biochim Biophys Acta 1247: 12–16. 7873580 10.1016/0167-4838(94)00166-e

[12] Hayashi T, Era S, Kawai K, Imai H, Nakamura K, et al (2000) Observation for redox state of human serum and aqueous humor albumin from patients with senile cataract. Pathophysiology 6: 237–243.

[13] Soejima A, Matsuzawa N, Hayashi T, Kimura R, Ootsuka T, et al (2004) Alteration of redox state of human albumin before and after hemodialysis. Blood Purif 22: 525–529. 15583477 10.1159/000082524

[14] Terawaki H, Yoshimura K, Hasegawa T, Matsuyama Y, Negawa T, et al (2004) Oxidative stress is enhanced in correlation with renal dysfunction: examination with the redox state of albumin. Kidney Int 66: 1988–1993. 15496170 10.1111/j.1523-1755.2004.00969.x

[15] Goldwasser P, Feldman J (1997) Association of serum albumin and mortality risk. J Clin Epidemiol 50: 693–703. 9250267 10.1016/s0895-4356(97)00015-2

[16] De Mutsert R, Grootendorst DC, Indemans F, Boeschoten EW, Krediet RT, et al (2009) Association between serum albumin and mortality in dialysis patients is partly explained by inflammation, and not by malnutrition. J Ren Nutr 19: 127–135. 19218039 10.1053/j.jrn.2008.08.003

[17] Djoussé L, Rothman KJ, Cupples LA, Levy D, Ellison RC (2002) Serum albumin and risk of myocardial infarction and all-cause mortality in the Framingham Offspring Study. Circulation 106: 2919–2924. 12460872 10.1161/01.cir.0000042673.07632.76

[18] Ogasawara Y, Mukai Y, Togawa T, Suzuki T, Tanabe S, et al (2007) Determination of plasma thiol bound to albumin using affinity chromatography and high-performance liquid chromatography with fluorescence detection: ratio of cysteinyl albumin as a possible biomarker of oxidative stress. J Chromatogr B Analyt Technol Biomed Life Sci 845: 157–163. 10.1016/j.jchromb.2006.08.006 16962833

[19] Danielski M, Ikizler TA, McMonagle E, Kane JC, Pupim L, et al (2003) Linkage of hypoalbuminemia, inflammation, and oxidative stress in patients receiving maintenance hemodialysis therapy. Am J Kidney Dis 42: 286–294. 12900810 10.1016/s0272-6386(03)00653-x

[20] Kalantar-Zadeh K, Kilpatrick RD, Kuwae N, McAllister CJ, Alcorn H Jr, et al (2005) Revisiting mortality predictability of serum albumin in the dialysis population: time dependency, longitudinal changes and population-attributable fraction. Nephrol Dial Transplant 20: 1880–1888. 15956056 10.1093/ndt/gfh941

[21] Amaral S, Hwang W, Fivush B, Neu A, Frankenfield D, et al (2008) Serum albumin level and risk for mortality and hospitalization in adolescents on hemodialysis. Clin J Am Soc Nephrol 3: 759–767. 18287254 10.2215/CJN.02720707 PMC2386701

[22] Himmelfarb J, McMonagle E (2001) Albumin is the major plasma protein target of oxidant stress in uremia. Kidney Int 60: 358–363. 11422772 10.1046/j.1523-1755.2001.00807.x

[23] Mera K, Anraku M, Kitamura K, Nakajou K, Maruyama T, et al (2005) Oxidation and carboxy methyl lysine-modification of albumin: possible involvement in the progression of oxidative stress in hemodialysis patients. Hypertens Res 28: 973–980. 16671336 10.1291/hypres.28.973

[24] Michelis R, Kristal B, Snitkovsky T, Sela S (2010) Oxidative modifications impair albumin quantification. Biochem Biophys Res Commun 401: 137–142. 20833127 10.1016/j.bbrc.2010.09.027

[25] Sogami M, Era S, Nagaoka S, Kuwata K, Kida K, et al (1985) High-performance liquid chromatographic studies on non-mercapt-mercapt conversion of human serum albumin. II. J Chromatogr 332: 19–27. 4055942 10.1016/s0021-9673(01)83283-0

[26] Tomida M, Ishimaru J, Hayashi T, Nakamura K, Murayama K, et al (2003) The redox states of serum and synovial fluid of patients with temporomandibular joint disorders. Jpn J Physiol 53: 351–355. 14975181 10.2170/jjphysiol.53.351

[27] Terawaki H, Takada Y, Era S, Funakoshi Y, Nakayama K, et al (2010) The redox state of albumin and serious cardiovascular incidence in hemodialysis patients. Ther Apher Dial 14: 465–471. 21175544 10.1111/j.1744-9987.2010.00841.x

[28] Sogami M, Era S, Nagaoka S, Kuwata K, Kida K, et al (1985) HPLC-studies on nonmercapt-mercapt conversion of human serum albumin. Int J Pept Protein Res 25: 398–402. 4019023 10.1111/j.1399-3011.1985.tb02191.x

[29] Cooper BA, Penne EL, Bartlett LH, Pollock CA (2004) Protein malnutrition and hypoalbuminemia as predictors of vascular events and mortality in ESRD. Am J Kidney Dis 43: 61–66. 14712428 10.1053/j.ajkd.2003.08.045

[30] Iseki K, Kawazoe N, Fukiyama K (1993) Serum albumin is a strong predictor of death in chronic dialysis patients. Kidney Int 44: 115–119. 8355451 10.1038/ki.1993.220

[31] Don BR, Kaysen G (2004) Serum albumin: relationship to inflammation and nutrition. Semin Dial 17: 432–437. 15660573 10.1111/j.0894-0959.2004.17603.x

[32] Oettl K, Stauber RE (2007) Physiological and pathological changes in the redox state of human serum albumin critically influence its binding properties. Br. J. Pharmacol. 151, 580–590. 10.1038/sj.bjp.0707251 PMC2013999 17471184

[33] Radi R, Beckman JS, Bush KM, Freeman BA (1991) Peroxynitrite oxidation of sulfhydryls. The cytotoxic potential of superoxide and nitric oxide. J Biol Chem 266: 4244–4250. 1847917

[34] Finch JW, Crouch RK, Knapp DR, Schey KL (1993) Mass spectrometric identification of modifications to human serum albumin treated with hydrogen peroxide. Arch Biochem Biophys 305: 595–599. 8373198 10.1006/abbi.1993.1466

[35] DeMaster EG, Quast BJ, Redfern B, Nagasawa HT (1995) Reaction of nitric oxide with the free sulfhydryl group of human serum albumin yields a sulfenic acid and nitrous oxide. Biochemistry 34: 11494–11499. 7547878 10.1021/bi00036a023

[36] Gatti RM, Radi R, Augusto O (1994) Peroxynitrite-mediated oxidation of albumin to the protein-thiyl free radical. FEBS Lett 348: 287–290. 8034056 10.1016/0014-5793(94)00625-3

[37] Thomas JA, Poland B, Honzatko R (1995) Protein sulfhydryls and their role in the antioxidant function of proteins-thiolation. Arch Biochem Biophys 319: 1–9. 7771771 10.1006/abbi.1995.1261

[38] Janatova J, Fuller JK, Hunter MJ (1968) The heterogeneity of bovine albumin with respect to sulfhydryl and dimer content. J Biol Chem 243: 3612–3622. 5690602

[39] Mera K, Anraku M, Kitamura K, Nakajou K, Maruyama T, et al (2005) The structure and function of oxidized albumin in hemodialysis patients: its role in elevated oxidative stress via neutrophil burst. Biochem Biophys Res Commun 334: 1322–1328. 16054887 10.1016/j.bbrc.2005.07.035

[40] Himmelfarb J, Stenvinkel P, Ikizler TA, Hakim RM (2002) The elephant in uremia: oxidant stress as a unifying concept of cardiovascular disease in uremia. Kidney Int 62: 1524–38. 12371953 10.1046/j.1523-1755.2002.00600.x

[41] Beddhu S, Kaysen GA, Yan G, Sarnak M, Agodoa L, et al (2002) Association of serum albumin and atherosclerosis in chronic hemodialysis patients. Am J Kidney Dis 40: 721–7. 12324906 10.1053/ajkd.2002.35679

[42] Kalantar-Zadeh K, Brennan ML, Hazen SL (2006) Serum myeloperoxidase and mortality in maintenance hemodialysis patients. Am J Kidney Dis 48: 59–68. 16797387 10.1053/j.ajkd.2006.03.047

[43] Roberts MA, Thomas MC, Fernando D, Macmillan N, Power DA, et al (2006) Low molecular weight advanced glycation end products predict mortality in asymptomatic patients receiving chronic haemodialysis. Nephrol Dial Transplant 21: 1611–1617. 16520354 10.1093/ndt/gfl053

[44] Ishii T, Ohtake T, Okamoto K, Mochida Y, Ishioka K, et al (2011) Serum biological antioxidant potential predicts the prognosis of hemodialysis patients. Nephron Clin Pract 117: c230–6. 20805696 10.1159/000320201

